# Factors associated with self-reports of limitations in activities of daily living among Medicare Fee-for-Service recipients

**DOI:** 10.1186/s12877-024-05242-4

**Published:** 2024-08-01

**Authors:** Ron D. Hays, Ann Haas, Amelia M. Haviland, Steven C. Martino, Nate Orr, Joy Binion, Marc N. Elliott

**Affiliations:** 1 RAND Corporation, 1776 Main Street, Santa Monica, CA 90407 US; 2 UCLA Department of Medicine, Division of General Internal Medicine & Health Services Research, Los Angeles, 1100 Glendon Avenue, CA 90024 US; 3 RAND Corporation, 4570 Fifth Ave, Pittsburgh, PA 15213 US; 4 Carnegie Mellon University, Hamburg Hall, 5000 Forbes Ave, Pittsburgh, PA 15213 US; 5 Centers for Medicare & Medicaid Services, 7500 Security Blvd, Baltimore, MD 21244 US

**Keywords:** Physical function, Physical limitations, Medicare, Fee-for-Service

## Abstract

**Background:**

Physical function is an important indicator of physical health and predicts mortality. This study identified characteristics associated with limitations in Medicare recipients' activities of daily living.

**Methods:**

2019 Consumer Assessment of Healthcare Providers and Systems Fee-for-Service Medicare Survey data: 79,725 respondents (34% response rate) who were 65 and older and 53% female; 7% Black, 5% Hispanic, 4% Asian American, Native Hawaiian, or other Pacific Islander, 2% Multiracial, 1% American Indian/Alaskan Native; 35% with high school education or less. Walking, getting in and out of chairs, bathing, dressing, toileting, and eating (scored as having no difficulty versus being able to do with difficulty or unable to do) and a scale of these items were regressed on patient characteristics.

**Results:**

After adjustment for all characteristics, function limitations were found for those who smoked (effect sizes of significant associations range .04-.13), had chronic health conditions (.02-.33), were 85 years or older (.09-.46), needed assistance completing the survey (.32–1.29), were female (.05-.07), and had low income and assets (.15-.47).

**Conclusions:**

These nationally representative U.S. estimates of physical function characteristics are useful for interventions for vulnerable population subgroups.

## Introduction

Physical functioning is the ability to conduct activities ranging from self-care to more challenging and vigorous activities that require increasing degrees of mobility, strength, or endurance [1]. A previous study of 366,701 adults in Medicare (managed care or Fee-for-Service) showed that a minority reported that they had difficulty or were unable to do six physical function activities: eating (6%), toileting (9%), dressing (12%), bathing (15%), getting in and out of chairs (22%), and walking (31%) [2].

Hardy et al. [3] found that the inability to walk one-quarter of a mile was associated with more hospitalizations, total annual healthcare costs, and mortality in adults with Medicare insurance in the U.S. Similarly, functional limitations are associated with greater hospitalizations and emergency department admissions among older Mexican Americans with Alzheimer’s disease and related dementias [4]. In addition, limitations in activities of daily living have been linked with a higher likelihood of unmet healthcare needs [5].

Smoking, health conditions, age, gender, race and ethnicity, and socioeconomic status may be associated with functional limitations. Smoking has been linked to worse physical function [6], but one study found no differences in aerobic or anaerobic physical fitness by smoker status among women [7]. Worse physical function has been consistently observed for those with more chronic health conditions [8, 9]. Prior studies also document more limitations with age, especially among older women [10–13]. Poorer physical function in African Americans than in White Women has been reported [14], but this may be partly due to socioeconomic differences [15]. Moreover, there is limited information about the physical function of other racial-and-ethnic groups. While less educational attainment is related to poorer physical function [10, 14], one study showed a lack of associations between physical functioning and neighborhood poverty, education, and income [16]. The inconsistent results of prior studies are partly due to small and unrepresentative samples of the older population.

An analysis of data from more than 170,280 enrollees in Medicare managed care included in the Surveillance, Epidemiology, and End Results (SEER) Medicare Health Outcomes Survey (MOS) data indicated worse self-reported physical health for Hispanic enrollees, Medicaid enrollees, and those with lower income and less education [17]. A subsequent analysis of the SEER-MHOS data indicated worse physical health for Hispanic and non-Hispanic Black enrollees than for non-Hispanic Asians/Pacific Islander and non-Hispanic White enrollees [18].

The SEER-MHOS data is limited to Medicare Advantage enrollees from the 13 SEER regions of the U.S. Identifying the characteristics of Fee-for-Service recipients throughout the U.S. who report physical functioning limitations is needed to target care for this vulnerable Medicare subgroup. Patient characteristics associated with physical function have not been reported for a large and representative sample of 65-year-old Fee-for-Service recipients. This paper examines associations of patient characteristics with each of six physical function items and an index of these items in a nationally representative sample of Medicare Fee-for-Service patients in the U.S.

## Materials and methods

The RAND Institutional Review Board approved this study. We analyzed survey data from the 2019 CAHPS Medicare Fee-for-Service Survey. Data were collected from March to May 2019 using a mixed-mode data collection protocol consisting of two survey mailings and telephone follow-up of non-respondents to the mailed questionnaire. The survey sample covers all 50 U.S. states, the District of Columbia, and Puerto Rico.

The survey asked about eating, using the toilet, dressing, bathing, getting in or out of chairs, and walking. These six items were administered using a common item stem: “Because of a health or physical problem, are you unable to do or have any difficulty doing the following activities?” Study participants were offered three response options: *I am unable to do this activity; Yes, I have difficulty; No, I do not have difficulty*. For the analyses, items were dichotomized (0 = have no difficulty; 1 = able to do with difficulty or unable to do). Support for the reliability and construct validity of these self-reports of limitations in activities of daily living were reported elsewhere [2].

The survey included important variables from prior literature and those used in standard CAHPS Medicare case-mix adjustment analyses. Assessed were race and ethnicity, educational attainment, and whether the respondent was a current smoker, lived alone, had a primary care doctor, and had proxy assistance completing the survey. In addition, the survey asked whether respondents had ever been diagnosed by a doctor with each of six conditions: angina or coronary heart disease; cancer other than skin cancer; emphysema, asthma, or COPD (chronic obstructive pulmonary disease); any diabetes or high blood sugar; hypertension or high blood pressure, and a heart attack. Age, gender, survey language, dual eligibility for Medicaid and Medicare, and eligibility for a low-income subsidy (LIS) for prescription drug coverage were available from the Centers for Medicare & Medicaid Services (CMS) or survey administrative records. Dual eligibility and LIS coverage are associated with having low income or assets.

The analytic sample was 79,725 adults 65 and older: 53% were female, 30% lived alone, and 8% were current smokers. Sample characteristics are summarized in Table 1.

**Table 1:** 2019 Medicare Fee-for-Service Consumer Assessment of Healthcare Providers and Systems (CAHPS) sample ages 65 and older (*n* = 79,725)

Characteristics	%
**Female**	53%
**Age**	
65-69	26%
70-74	28%
75-79	19%
80-84	13%
85+	14%
**Race and Ethnicity**	
American Indian/Alaskan Native	1%
Asian American and Native Hawaiian or other Pacific Islander	4%
Black	7%
Hispanic	5%
Multiracial (i.e., more than one race selected)	2%
White	82%
**Current Smoker**	8%
**Health Conditions**	
Angina	15%
Cancer	18%
Chronic obstructive pulmonary disease	15%
Diabetes	26%
Hypertension	64%
Heart attack	10%
**Education**	
Some high school or less	9%
High school or General Education Diploma	26%
Some college or 2-year degree	28%
4-year degree	15%
More than a 4-year degree	21%
**Financial assistance**	
Neither dual eligibility nor receipt of low-income subsidy	90%
Low-income subsidy only	1%
Dual eligibility, with or without low-income subsidy	8%
**Live alone**	30%
**Survey Language**	
English	98%
Spanish	2%
Chinese	<1%
**Have a primary doctor**	94%
**Proxy Status**	
No proxy assistance	90%
Proxy helped, but did not answer for the patient	6%
Proxy answered for the patient	4%

Age, financial assistance (dual eligibility and receipt of a low-income subsidy), survey language, and gender were obtained from the Centers for Medicare & Medicaid Services or survey administrative records, and all cases had complete data. All other variables were derived from the survey and were missing for some cases; rates of item missingness were 6% for education, 4% for race and ethnicity, 3% for living alone, 11% for proxy status, 4-5% for the six chronic conditions, 5% for smoking status, and 2% for having a primary doctor

### Analysis plan

We hypothesized that older age, having health conditions, dual eligibility, LIS coverage, and requiring proxy assistance completing the survey would be significantly associated with worse physical function. We report frequencies for the six physical function items and estimate multivariate ordinary least squares (OLS) regression models with physical function items and the simple-summated six-item scale as dependent variables. For ease of interpretation, we dichotomized items into no difficulty versus some difficulty or unable to do. OLS regressions are robust to non-normality, including dichotomization at these sample sizes, and support tests of means and correlations [19]. The scale was calculated as the mean across the six items; each scored 0 = no difficulty, 50 = some difficulty, and 100 = unable to do (coefficient alpha = 0.94). The six items and the scale were z-scored (mean = 0; standard deviation = 1) before use in modeling; coefficients can be interpreted as effect sizes. We report the statistical significance and 95% confidence intervals for the coefficients.

Models adjust for coverage type (Fee-for-Service with versus without a Part D Plan); Census Division (New England, Middle Atlantic, East North Central, West North Central, South Atlantic, East South Central, West South Central, Mountain, Pacific) or Puerto Rico; and rurality (residence in a metropolitan division or metropolitan statistical area, a micropolitan statistical area, or outside of metropolitan or micropolitan areas).

The data were weighted to represent the population of Fee-for-Service recipients age 65 years or older using the probability of sampling and response. Analyses were conducted using SAS 9.4 (TS1M2).

## Results

Of the 238,051 people sampled for this survey, 1,363 (1%) were ineligible, and 156,963 (66%) were non-respondents, resulting in a 34% response rate. The percentage of the sample reporting limitations (any difficulty or inability to do the activity) was 5% for eating, 8% for toileting, 11% for dressing, 13% for bathing, 19% for getting in and out of chairs, and 27% for walking. These rates are comparable to those collected a decade ago with the same items in another sample of adults with Medicare insurance [2].

Table 2 summarizes the results of the multivariate regression models. Females were more likely than males to report limitations in bathing, getting in and out of chairs, walking, and the physical function scale. Older age was associated with more limitations for all six activities and the scale score, with limitations being most common among the 85 and older group. Multiracial individuals reported the most limitations of any racial-and-ethnic group, and Asian American and Native Hawaiian or other Pacific Islander respondents reported the fewest. Current smokers and those with chronic health conditions were more likely to report physical limitations.

**Table 2:** Linear regression of limitations in six physical function items and six-item scale on patient characteristics in the 2019 Medicare CAHPS Fee-for-Service Survey, ages 65 and older

	Bathing	Dressing	Eating	Chairs	Walking	Toileting	Scale
**Female**	.05 (.03, .07)***	.00 (-.02, .01)	.01 (-.01, .03)	.05 (.04, .07)***	.07 (.05, .08)***	.01 (-.01, .03)	.03 (.01, .05)***
**Age**							
70–74	-.01 (-.03, .01)	-.01 (-.03, .01)	-.01 (-.03, .01)	-.01 (-.03, .01)	-.01 (-.03, .01)	-.02 (-.04, .00)	-.02 (-.04, .00)
75–79	.03 (.01, .05)**	.02 (.00, .05)	.01 (-.02, .03)	.06 (.03, .08)***	.08 (.05, .10)***	.03 (.01, .06)**	.04 (.01, .06)**
80–84	.09 (.06, .12)***	.08 (.05, .11)***	.03 (< .01, .06)*	.14 (.11, .16)***	.18 (.16, .21)***	.06 (.03, .09)***	.09 (.06, .11)***
85 +	.35 (.31, .38)***	.25 (.22, .28)***	.09 (.06, .12)***	.37 (.34, .40)***	.46 (.44, .49)***	.21 (.18, .24)***	.28 (.25, .31)***
**Race-and-ethnicity**							
Asian American and Native Hawaiian or other Pacific Islander	-.15 (-.20, -.10)***	-.11 (-.16, -.06)***	-.04 (-.10, .01)	-.20 (-.25, -.15)***	-.17 (-.22, -.13)***	-.07 (-.13, -.02)**	-.15 (-.20, -.10)***
American Indian/Alaska Native	.02 (-.12, .15)	.02 (-.11, .16)	.12 (-.03, .27)	.05 (-.07, .17)	.11 (-.01, .22)	-.01 (-.14, .12)	.05 (-.08, .18)
Black	.04 (.00, .08)	.04 (.00, .08)	.00 (-.04, .04)	.04 (< .01, .08)*	.06 (.02, .10)**	.03 (-.01, .07)	.01 (-.02, .05)
Hispanic	-.01 (-.06, .04)	.05 (-.01, .10)	.05 (.00, .11)	-.06 (-.10, -.01)*	-.04 (-.08, .01)	.02 (-.03, .08)	.00 (-.05, .06)
Multiracial	.20 (.12, .28)***	.13 (.05, .21)**	.10 (.02, .19)*	.17 (.09, .24)***	.23 (.15, .30)***	.08 (< .01, .17)*	.09 (.04, .15)**
**Current Smoker**	.04 (.01, .08)**	.02 (-.02, .05)	.05 (.01, .08)*	.04 (< .01, .07)*	.13 (.10, .16)***	.03 (.00, .06)	.05 (.01, .08)**
**Health Conditions**							
Angina	.11 (.08, .14)***	.08 (.05, .11)***	.03 (< .01, .06)*	.12 (.09, .14)***	.17 (.14, .19)***	.06 (.03, .08)***	.09 (.06, .11)***
Cancer	.05 (.03, .07)***	.04 (.02, .06)***	.03 (.01, .06)**	.08 (.06, .10)***	.08 (.06, .09)***	.02 (.00, .04)	.04 (.02, .06)***
Chronic obstructive pulmonary disease	.23 (.20, .26)***	.17 (.15, .20)***	.08 (.05, .10)***	.22 (.19, .24)***	.33 (.31, .35)***	.11 (.08, .14)***	.18 (.16, .21)***
Diabetes	.14 (.12, .16)***	.11 (.09, .14)***	.06 (.04, .08)***	.20 (.18, .22)***	.23 (.22, .25)***	.10 (.08, .12)***	.15 (.13, .16)***
Hypertension	.03 (.02, .05)***	.02 (.01, .04)**	.00 (-.02, .02)	.09 (.08, .11)***	.14 (.12, .15)***	.02 (< .01, .04)*	.05 (.03, .07)***
Heart attack	.09 (.06, .13)***	.11 (.08, .15)***	.08 (.04, .11)***	.12 (.09, .16)***	.14 (.11, .17)***	.09 (.06, .13)***	.12 (.08, .15)***
**Education**							
< = 8th grade	-.03 (-.10, .04)	-.08 (-.15, > -.01)*	-.01 (-.09, .07)	-.11 (-.17, -.04)***	-.08 (-.14, -.02)**	-.03 (-.11, .05)	-.07 (-.14, > -.01)*
Some high school	-.03 (-.08, .01)	-.07 (-.12, -.02)**	-.09 (-.14, -.04)***	-.04 (-.09, .00)	.01 (-.04, .05)	-.07 (-.12, -.02)**	-.08 (-.12, -.03)***
Some college	.02 (.00, .04)	.02 (.00, .04)	.00 (-.02, .03)	.00 (-.02, .02)	-.02 (.04, .00)	.00 (.02, .03)	.00 (-.02, .02)
4-year degree	-.03 (-.06, -.01)**	-.02 (-.04, .01)	-.03 (-.05, .00)	-.06 (-.08, -.03)***	-.09 (-.12, -.07)***	-.02 (-.04, .01)	-.05 (-.08, -.03)***
> 4-year degree	-.05 (-.07, -.02)***	-.03 (-.05, > -.01)*	-.04 (-.07, -.02)***	-.08 (-.10, -.05)***	-.12 (-.14, -.10)***	-.02 (-.05, > -.01)*	-.07 (-.09, -.05)***
**Financial assistance**							
Low-income subsidy, but not dually eligible	.26 (.17, .36)***	.16 (.07, .26) ***	.17 (.07, .27) **	.25 (.16, .34)***	.32 (.23, .40)***	.15 (.05, .24)**	.19 (.11, .28)***
Dual eligibility, with or without low-income subsidy	.47 (.42, .52)***	.41 (.36, .46)***	.26 (.21, .32)***	.35 (.30, .39)***	.42 (.38, .46)***	.30 (.24, .35)***	.35 (.31, .40)***
**Live alone**	.02 (.00, .04)	.00 (-.02, .01)	.00 (-.02, .01)	.02 (<.01, .04)*	.04 (.02, .05)***	-.01 (-.03, .01)	.00 (-.02, .01)
**Survey Language**							
Spanish	-.04 (-.17, .09)	.00 (-.14, .14)	-.07 (-.22, .08)	.15 (.03, .28)*	-.01 (-.12, .10)	.01 (-.13, .16)	-.05 (-.17, .06)
Chinese	-.45 (-1.10, .20)	-.08 (-.85, .69)	.36 (-.68, 1.40)	-.25 (-.87, .38)	.19 (-.34, .72)	.12 (-.75, .99)	-.12 (-.65, .41)
**Proxy**							
Helped but did not answer	.66 (.60, .71)***	.67 (.61, .73)***	.32 (.26, .38)***	.49 (.44, .53)***	.42 (.38, .47)***	.53 (.47, .59)***	.51 (.46, .56)***
Answered for patient	1.29 (1.23, 1.36)***	1.27 (1.20, 1.34)***	1.04 (.94, 1.13)***	.87 (.81, .92)***	.71 (.66, .76)***	1.21 (1.13, 1.29)***	1.23 (1.15, 1.30)***
**Have a primary doctor**	-.02 (-.05, .02)	-.02 (-.05, .01)	-.05 (-.08, -.01)*	-.01 (-.04, .02)	.02 (-.01, .05)	-.03 (-.07, .00)	-.02 (-.05, .02)

Cells are Estimate (95% Confidence Interval) *p<.05 **p<.01 ***p<.001. For statistically significant estimates where one end of the confidence interval rounds to 0.00, we use <0.01 and >-0.01 to indicate positive or negative coefficients, respectively

Missingness rates were 4% for Bathing, 4% for Dressing, 5% for Eating, 4% for Chairs, 4% for Walking, 4% Toileting, and 6% for the scale. The physical function items were z-scored (mean=0; standard deviation=1; higher score indicates some difficulty or unable to do the activity) before use in modeling; coefficients can be interpreted as effect sizes. The scale was calculated as the mean across the six items; each scored as 0=no difficulty, 50=some difficulty, 100=unable to do. The scale was z-scored (mean=0; standard deviation=1) before use in modeling; coefficients can be interpreted as effect sizes

Models also adjust for coverage type (Fee-for-Service with versus without a Part D Plan); Census Division (New England, Middle Atlantic, East North Central, West North Central, South Atlantic, East South Central, West South Central, Mountain, Pacific) or Puerto Rico; and rurality (residence in a metropolitan division or metropolitan statistical area, a micropolitan statistical area, or outside of metropolitan or micropolitan areas)

The holdout groups in the model were age 65-69, high school or general education diploma, no subsidy or dual eligibility, English, White, male, do not live alone, no proxy, no chronic conditions, not a current smoker, and do not have a primary doctor

There was a non-monotonic relationship between educational attainment and physical limitations, as the highest level of limitations was seen in those with a high school degree or some college. Respondents with dual eligibility or who received the LIS were likelier to report physical limitations than those who were neither dually eligible nor LIS recipients. Those living alone reported more limitations in getting in and out of chairs and walking than those living with others. Patients needing proxy respondents reported substantially more physical limitations. Patients with a primary care doctor were less likely to report limitations in eating.

## Discussion

This U.S. Medicare Fee-for-Service sample analysis identified several significant correlates of activities of daily living in those 65 and older. Smokers, people with chronic health conditions, older adults, those who required help completing the survey, females, Multiracial people, and those with limited income and assets were significantly more likely to report limitations in physical functioning.

These results from a representative sample of Medicare Fee-for-Service recipients in the U.S. are important because of the unique findings related to race/ethnic and socioeconomic variables. Prior work compared Black, Hispanic, and White respondents but did not include a multiracial category [17, 18]. The current study found more limitations on all six physical function items for multiracial adults than non-Hispanic White respondents. The study also provides new information about the role of low income in functional status, as there were unique associations of receiving LIS with more impairment in all six activities of daily living. The study also found that dually eligible individuals and those requiring proxy assistance to complete the survey were significantly more likely to report limitations for all six physical functioning items.

Some prior studies have found that lower education is associated with more functional limitations. However, education is considered a surrogate for socioeconomic position. In our primary regression models, we adjusted for LIS, a measure of limited income and assets. We found negative regression coefficients (indicating less functional limitations) for those who did not graduate from high school and those with a 4-year or higher college degree compared to those who only graduated from high school. Education was negatively associated with functional limitations in models that adjusted for only LIS and only for proxy (not reported). Hence, the total effect of education on functional limitations and the indirect effects of education through LIS and proxy were negative, but the direct effect was quadratic. Future studies are needed to evaluate education's direct and indirect effects on functional limitations.

Among the chronic conditions measured in the study, chronic obstructive pulmonary disease and diabetes tended to have the most consistent and substantial unique associations with lower physical function. A previous analysis of the Surveillance, Epidemiology, and End Results Medicare Health Outcomes Survey of 126,366 adults 65 and older found similar significant unique associations of those chronic conditions with the SF-6D health-related quality of life preference measure [20]. The greater level of limitations in physical functioning reported by women (bathing, chairs, walking) and older individuals (all six activities) is consistent with other studies [11–15]. In addition, the finding that current smokers reported more limitations in getting in and out of chairs, walking, and eating than non-smokers is consistent with a prior study [8]. Moreover, Black respondents reported more limitations in getting in and out of chairs and walking than White respondents.

This study has limitations that are important to acknowledge. One limitation is the focus on only Medicare Fee-for-Service. Results may not generalize to Medicare recipients enrolled in Medicare Advantage (managed care plans). However, prior studies [3, 18] have included this subgroup of the Medicare population. In addition, the study relies on self-reports, and some of the differences in this study could be partly due to differential item function (DIF). For example, Teresi et al. [21] found that Functional Assessment in Acute Care MCAT mobility items related to stairs had noteworthy DIF and recommended that these items not be included in short forms of the measure. While we do not include this item, research is needed to evaluate the measurement equivalence of the six physical items examined in the study by age, gender, race and ethnicity, and educational attainment.

The nature of the items included in the study is another potential study limitation. Most of the sample reported no limitations on the six physical function items. Results could differ for items representing higher levels of physical function, such as: “Does your health now limit you from doing vigorous activities, such as running, lifting heavy objects, or participating in strenuous sports?” In addition, we did not assess all health conditions associated with functional limitations, such as arthritis and dementia. Another limitation of our study is the 34% response rate. However, the response rate is a weak proxy for non-response bias [22], and modest differences between responders and non-responders on observed characteristics were accounted for by non-response weighting [23].

In summary, this large and nationally representative sample of Medicare Fee-for-Service recipients provides important information about individual characteristics associated with limitations in physical function. This information is useful for identifying subgroups of older individuals in the U.S. who can be targeted for monitoring and interventions to prevent falls and other negative consequences [24]. It is also important to recognize the notable variation in daily living activities among those in the higher-risk categories [25].

## Data Availability

The datasets generated and analyzed during the current study are not publicly available due to restrictions on access by the United States Centers for Medicare & Medicaid Services.
